# The Phylogenetic Diversity of Metagenomes

**DOI:** 10.1371/journal.pone.0023214

**Published:** 2011-08-31

**Authors:** Steven W. Kembel, Jonathan A. Eisen, Katherine S. Pollard, Jessica L. Green

**Affiliations:** 1 Institute of Ecology and Evolution, University of Oregon, Eugene, Oregon, United States of America; 2 University of California Davis Genome Center, Department of Evolution and Ecology and Medical Microbiology and Immunology, University of California Davis, Davis, California, United States of America; 3 Gladstone Institutes, Institute for Human Genetics and Division of Biostatistics, University of California San Francisco, San Francisco, California, United States of America; 4 Santa Fe Institute, Santa Fe, New Mexico, United States of America; University of Wyoming, United States of America

## Abstract

Phylogenetic diversity—patterns of phylogenetic relatedness among organisms in ecological communities—provides important insights into the mechanisms underlying community assembly. Studies that measure phylogenetic diversity in microbial communities have primarily been limited to a single marker gene approach, using the small subunit of the rRNA gene (SSU-rRNA) to quantify phylogenetic relationships among microbial taxa. In this study, we present an approach for inferring phylogenetic relationships among microorganisms based on the random metagenomic sequencing of DNA fragments. To overcome challenges caused by the fragmentary nature of metagenomic data, we leveraged fully sequenced bacterial genomes as a scaffold to enable inference of phylogenetic relationships among metagenomic sequences from multiple phylogenetic marker gene families. The resulting metagenomic phylogeny can be used to quantify the phylogenetic diversity of microbial communities based on metagenomic data sets. We applied this method to understand patterns of microbial phylogenetic diversity and community assembly along an oceanic depth gradient, and compared our findings to previous studies of this gradient using SSU-rRNA gene and metagenomic analyses. Bacterial phylogenetic diversity was highest at intermediate depths beneath the ocean surface, whereas taxonomic diversity (diversity measured by binning sequences into taxonomically similar groups) showed no relationship with depth. Phylogenetic diversity estimates based on the SSU-rRNA gene and the multi-gene metagenomic phylogeny were broadly concordant, suggesting that our approach will be applicable to other metagenomic data sets for which corresponding SSU-rRNA gene sequences are unavailable. Our approach opens up the possibility of using metagenomic data to study microbial diversity in a phylogenetic context.

## Introduction

In recent years there have been significant advances in the development of phylogenetic diversity statistics to quantify the relative importance of processes such as dispersal, competition and environmental filtering in shaping community structure [1], [2]. These tools have been applied to the study of microbial communities in ecosystems ranging from mountains to ocean depths [3]–[7], and within hosts including the human microbiome [8] and plant phyllosphere [9]. While these studies have provided great insight into the processes responsible for microbial diversity, they have almost exclusively used a single gene, the 16S SSU-rRNA gene [10], as a phylogenetic marker to study microbial community structure [3], [4], [8], [11], [12].

As sequencing costs have declined and novel technologies developed, a new field has emerged in the study of microbial communities wherein DNA isolated from environmental samples is randomly sequenced using the same shotgun approaches used to sequence the human and other genomes [13]. This metagenomic sequencing offers many advantages when studying microbial diversity [14], including the potential to provide insights into the ecological distribution of multiple gene families simultaneously. Metagenomic data also allows one to sample a broad diversity of genes at once, rather than focusing on one (e.g., SSU-rRNA) or a few genes. SSU-rRNA genes, though very powerful, are not perfect indicators of phylogenetic relatedness [15], and variance in copy number between taxa makes SSU-rRNA genes less than ideal for assessing relative abundance patterns [16]. The non-targeted nature of shotgun sequencing allows a more representative sample of entire communities than can be obtained using targeted methods such as PCR amplification [13], [16], although metagenomic studies are not without their own biases, including the fact that not all genes or clades can be sequenced equally well by metagenomic techniques [17].

The decreasing cost of sequencing technologies will lead to a massive increase in the sequencing depth and overall availability of metagenomic data [13], [18], [19]. Complete microbial genomes will also become increasingly easy to sequence [20], which in turn will allow improved alignment, taxonomic identification, and phylogenetic placement of metagenomic reads from multiple gene families. Despite the promise of metagenomic data to provide insights into microbial ecology and evolution, methods to measure phylogenetic diversity based on metagenomic data remain in their infancy [21]. Previous studies of microbial diversity using metagenomic data have generally quantified the structure of microbial assemblages by binning metagenomic sequences into taxonomically or functionally similar groups based on overall sequence similarity [22], [23], or on single marker genes [24], and to date it has been challenging to apply phylogenetic diversity statistics to metagenomic data sets. To address this challenge, we present a novel approach for inferring phylogenetic relationships among assemblages of microorganisms based on metagenomic data, and apply this method to illuminate patterns of microbial phylogenetic diversity along an oceanic depth gradient [22].

## Results and Discussion

### Phylogenetic inference from metagenomic data

To study the phylogenetic diversity of microbial communities, one needs to first generate hypotheses regarding the phylogenetic relationships among the organisms in those communities. While in theory metagenomics has enormous potential for such studies, in practice making use of metagenomic data to quantify phylogenetic diversity has been challenging. A key challenge is which gene or genes to study. While previous studies have constructed phylogenetic trees for single genes based on metagenomic data [16], [24], this approach uses only a small fraction of the data available from metagenomic sampling. Another challenge relates to the fragmented nature of reads produced by shotgun sequencing of environmental samples, which results in many reads being mutually non-overlapping, making estimation of the phylogenetic distance among those reads difficult.

**Figure 1:**
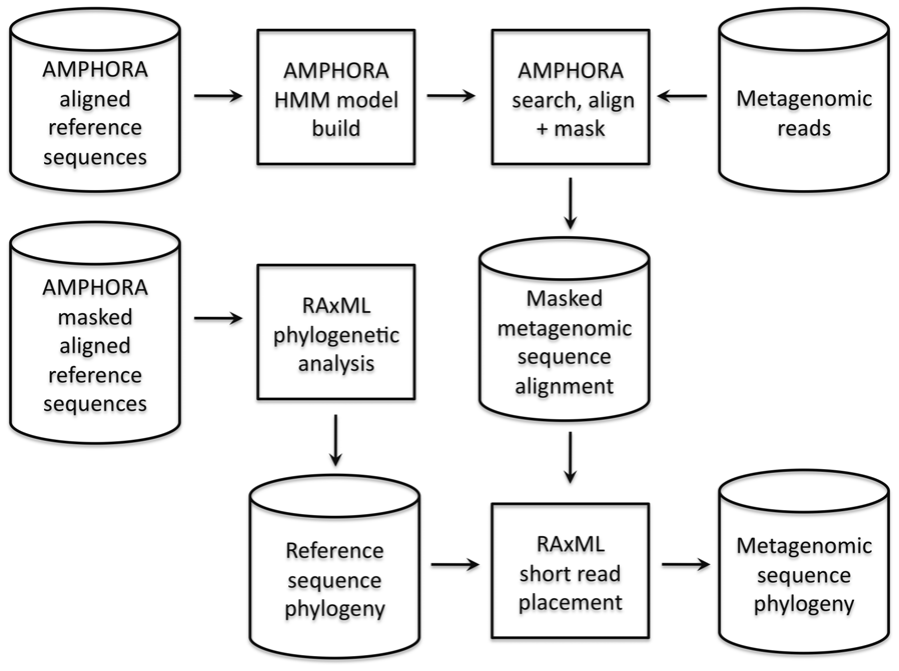
Conceptual overview of approach to infer phylogenetic relationships among sequences from metagenomic data sets.

To overcome these challenges, we took advantage of the rapidly increasing availability of fully sequenced bacterial genomes [20]. Specifically, we used full-length gene sequences from these genomes as a phylogenetic scaffold to allow inference of phylogenetic relationships among metagenomic sequences from different gene families (Figure 1). This approach extends and unifies the approaches used by existing studies of phylogenetic relationships among metagenomic reads, which have generally focused on discovering novel functional gene families in metagenomic data sets based on individual genes [24], [25] or on phylogenetically-informed taxonomic identification of metagenomic reads [16], [26]–[28].

**Figure 2:**
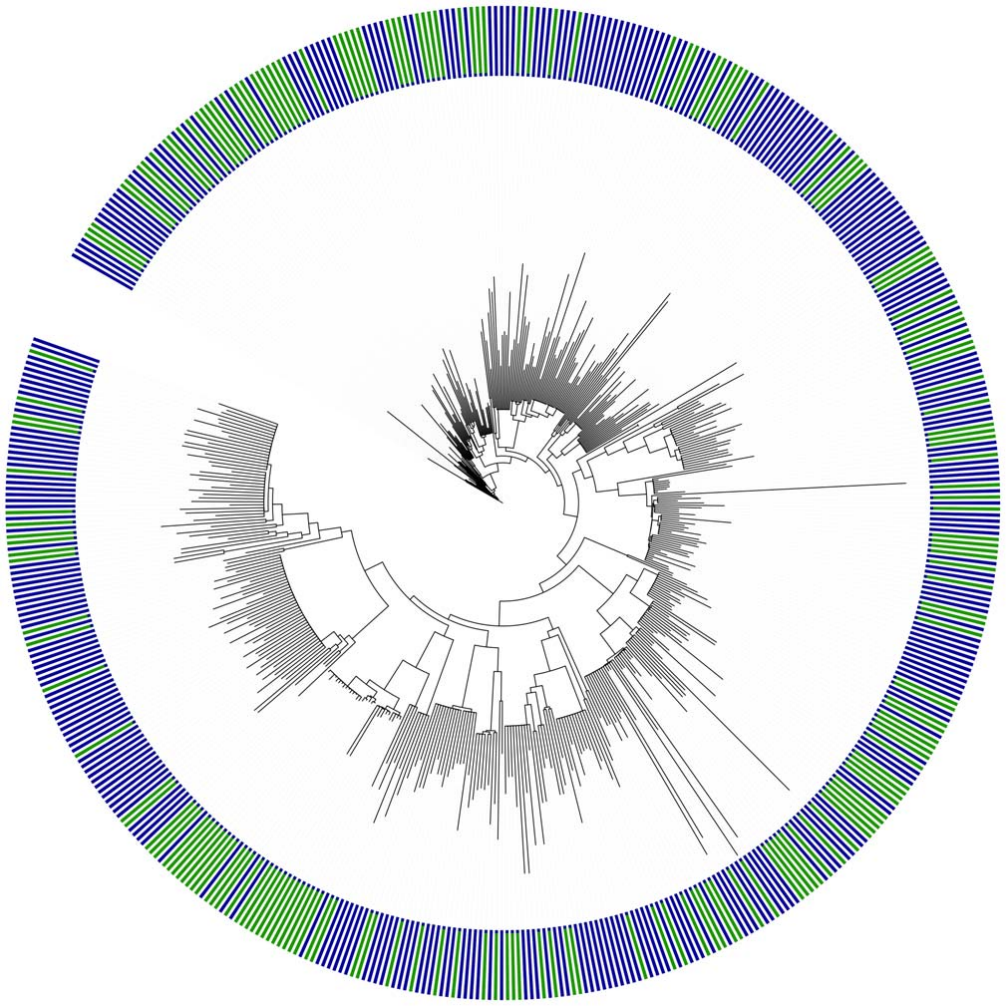
Phylogenetic tree linking metagenomic sequences from 31 gene families along an oceanic depth gradient at the HOT ALOHA site [22]. The depth from which sequences were collected is indicated by bar color (green = photic zone (<200 m depth), blue = nonphotic zone). The displayed tree is the one that was identified as having the maximum likelihood by placing metagenomic reads on a reference phylogeny inferred with a WAG + G model partitioned by gene family in RAxML [29]. The phylogeny is arbitrarily rooted at *Thermus* for display purposes.

For our analysis, we began with a set of 31 gene families (which we refer to as ‘marker’ genes) chosen based on their universality, low copy number, phylogenetic signal, and low rates of horizontal gene transfer [26]. We built alignments and inferred a phylogeny linking the sequences from these marker gene families across 571 fully sequenced bacterial genomes (which we refer to as ‘reference’ sequences). We then derived marker gene models from the reference sequences and employed the AMPHORA bioinformatics pipeline [26] to identify metagenomic sequences belonging to each marker gene family in the seven environmental samples of the Hawaii Ocean Time-series (HOT) ALOHA station data set [22]. Next, we aligned the metagenomic sequences to the gene models and placed each of them on the reference phylogeny using maximum likelihood short-read-placement methods [29] to account for variation in evolutionary rates across sites and gene families (Figure 2). Because these marker genes are almost exclusively single-copy genes [26], we expect to sample sequences from organisms in the environment in proportion to their relative abundance. Following this assumption, this approach allowed us to measure diversity directly from individual sequences rather than binning them into taxonomic groups or OTUs.

### Measuring phylogenetic diversity using metagenomic data

We next evaluated the ability of our phylogenetic marker gene approach to detect patterns of diversity along the HOT ALOHA ocean depth gradient, and compared our approach to existing approaches to analyzing microbial data, including SSU-rRNA-gene-based measures of phylogenetic diversity and taxonomic composition. By analysis of phylogenetic relatedness among metagenomic sequences from the 31 marker gene families (Figure 2), we measured phylogenetic diversity within each environmental sample as the mean pairwise phylogenetic distance (*MPD*) separating all pairs of sequences in the sample [1], [30]. As with most phylogenetic diversity metrics, this widely-used measure of phylogenetic diversity is correlated with the number of sequences present in a sample [2], [31], [32]. Since both sampling intensity and the number of metagenomic sequences identified varied among samples, we standardized the observed phylogenetic diversity in each sample by comparing it to the values expected from 999 random draws of an equal number of sequences from the pool of all metagenomic reads to calculate a standardized effect size (*SES*) of phylogenetic diversity [33]:
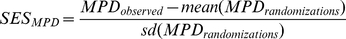



The resulting standardized phylogenetic diversity measure (*SES_MPD_*) expresses how different the observed phylogenetic diversity value is (in units of standard deviations (*sd*)) from the average (*mean*) phylogenetic diversity in the randomly generated communities. Positive values of *SES_MPD_* indicate phylogenetic evenness (co-occurring sequences more phylogenetically distantly related than expected by chance), while negative values indicate phylogenetic clustering (co-occurring sequences more closely related than expected by chance).

**Figure 3:**
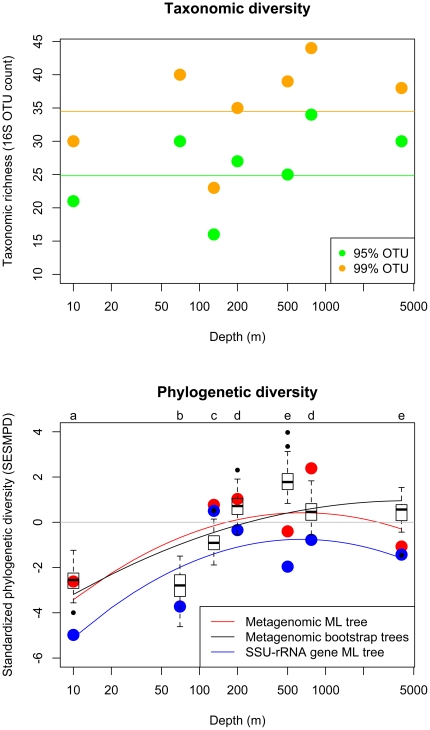
Taxonomic diversity and standardized phylogenetic diversity versus depth in environmental samples along an oceanic depth gradient at the HOT ALOHA site. axonomic diversity is calculated as OTU richness (number of OTUs) based on binning of SSU-rRNA gene sequences into OTUs at a 95% and 99% similarity cutoff. Phylogenetic diversity is calculated as the standardized effect size of the mean pairwise phylogenetic distances (*SES_MPD_*) among SSU-rRNA gene sequences (blue symbols) and metagenomic sequences from the 31 AMPHORA gene families (red symbols). Standardized phylogenetic diversity values less than zero indicate phylogenetic clustering (sequences more closely related than expected); values greater than zero indicate phylogenetic evenness (sequences more distantly related than expected). Phylogenetic diversity was estimated from the maximum likelihood phylogenies for SSU-rRNA gene and metagenomic data, as well as for 100 replicate phylogenies inferred from the metagenomic data with a phylogenetic bootstrap (black symbols). Lines indicate best-fit from quadratic regressions of diversity versus depth; the slopes of regressions of taxonomic diversity versus depth were not significantly different than zero (P>0.05). At all depths, standardized phylogenetic diversity across 100 bootstrap phylogenies differed significantly from the null expectation of zero (t-test, *P*<0.05). Phylogenetic diversity based on the 100 bootstrap phylogenies differed significantly among samples that do not share a letter label at the top of the panel (Tukey's HSD test, *P*<0.05).

Standardized phylogenetic diversity peaks at intermediate oceanic depth, with the lowest phylogenetic diversity in the shallowest samples (Figure 3). Phylogenetic diversity in the deepest samples is slightly less than at intermediate depth samples. This trend was observed for phylogenetic diversity calculated based on both PCR-derived SSU-rRNA gene sequences and metagenomic marker sequences, indicating that comparable results can be obtained from both types of sequence data. Phylogenetic diversity calculated for metagenomic sequences indicated that compared to a null model of drawing the observed number of sequences in each sample randomly from the entire phylogeny, samples from the photic zone (<200 m depth) were phylogenetically clustered (*SES_MPD_*<0), meaning that the sequences were more closely related than expected by chance. In contrast, samples from intermediate depths were phylogenetically even (*SES_MPD_*>0), meaning that the sequences were more distantly related than expected by chance. The deepest sample from 4000 m did not show a clear difference from the null model; it was either phylogenetically clustered or even relative to this null model depending on the method used to measure phylogenetic relatedness (SSU-rRNA gene and metagenomic ML phylogeny: *SES_MPD_*<0; metagenomic bootstrap phylogenies: *SES_MPD_*>0). Phylogenetic diversity calculated for SSU-rRNA gene sequences showed a similar unimodal pattern with highest diversity at intermediate depths, although SSU-rRNA gene phylogenetic diversity within samples was phylogenetically clustered relative to the null expectation at all depths.

The non-random phylogenetic diversity we observed along the depth gradient provides evidence for the role of different niche-based community assembly processes structuring these microbial communities. The phylogenetic clustering of sequences in the shallowest and deepest samples is consistent with the pattern expected if closely related species are ecologically similar [34], and the environment in these habitats select for a subset of bacteria which are able to survive in the relatively stressful conditions in these two habitats [1], [35]. This pattern is in line with predictions that the extremes of disturbance and resource availability gradients should select for a limited subset of taxa that possess the traits that allow them to survive in those habitats. In the case of the oceanic depth gradient, these extremes reflect the turbid and high-resource-availability photic zone, versus the low-resource-availability abyssal zone.

### Phylogenetic diversity makes sense of conflicting patterns of taxonomic diversity along depth gradients

Our approach provides a phylogenetic framework that makes sense of the inconsistent results of previous studies of microbial diversity along oceanic depth gradients. Studies using fingerprinting technologies such as T-RFLP to measure microbial diversity along depth gradients have found inconsistent results ranging from increasing to decreasing diversity with depth [36], [37]. Similarly, a recent study using pyrosequencing of SSU-rRNA gene PCR products from the HOT ALOHA transect [38] found differences in patterns of diversity with depth for different domains of microbial life and depending on the sequence similarity cutoff used to define OTUs. The unimodal relationship between bacterial phylogenetic diversity and depth we observed was predominantly driven by the lower phylogenetic diversity in samples at depths of 10 m and 70 m relative to deeper samples. The deepest samples showed phylogenetic diversity only slightly lower than the intermediate depth samples (Figure 3), although the deepest samples were phylogenetically clustered while the intermediate depth samples were phylogenetically overdispsersed based on our null-model analyses. These findings, which are based on a phylogenetic approach, provide a framework for interpreting the results of a recent SSU-rRNA pyrosequencing-based taxonomic diversity study at the same site by Brown *et al.* [38]. They found that when a high OTU similarity cutoff was used (100% or 98%), bacterial diversity decreased with depth, whereas when a lower similarity cutoff was used to define OTUs (80%), bacterial diversity increased with depth. Phylogenetic diversity can explain this pattern; we found that the shallowest samples were phylogenetically clustered, dominated by sequences from a few closely related clades. Thus, these samples should contain taxonomically similar organisms at a high similarity cutoff, and the high OTU diversity at a 100% or 98% cutoff in shallow waters is driven by the presence of numerous very closely related taxa at those depths. But an 80% OTU cutoff shows an increase in diversity with depth due to organisms from distantly related clades dominating communities at greater depths. In other words, shallow waters are occupied by a group of very closely related microbial taxa, whereas deeper waters contain a broader range of more distantly related taxa.

Phylogenetic diversity measures have commonly been applied in the analysis of SSU-rRNA data, but not for metagenomic data sets. A phylogenetic approach to measuring diversity from metagenomic data enables the detection of environmental diversity patterns that could be missed by commonly used methods that bin metagenomic sequences into OTUs or other taxonomic groupings. Since taxonomic binning methods estimate diversity using the number of distinct taxa in each community, they can provide similar measures of diversity for a sample of related versus the same number of divergent taxa. In other words, when the phylogenetic relatedness of taxa varies across communities, taxonomic binning methods may fail to detect this variation and will be sensitive to the choice of threshold for identifying distinct taxa. An added benefit of the phylogenetic approach is that it avoids the issue of choosing a similarity threshold to define OTUs or other ecologically relevant taxonomic groups, which can be extremely challenging in microbial diversity studies [39].

### Phylotyping using SSU-rRNA gene versus metagenomic marker genes

In addition to analyses of phylogenetic diversity, we examined variation in community composition with depth based on phylotyping of sequences using a phylogenetic framework. The AMPHORA bioinformatics pipeline performs phylotyping, which uses phylogenetic placement of the metagenomic reads to identify the taxonomic groups to which they belong. This approach is conceptually similar to existing tools for taxonomic classification of metagenomic sequences (e.g. MEGAN [40]), with important differences. First, it makes use of phylogenetic trees and placement of sequences on these trees under a quantitative evolutionary model rather than surrogates for phylogeny (e.g., BLAST similarity scores). Second, AMPHORA focuses on analyzing a set of phylogenetic marker genes chosen for their utility in phylogenetic classification, while other binning methods generally make use of sequences from many gene families, not all of which are phylogenetically informative, when binning sequences.

**Figure 4:**
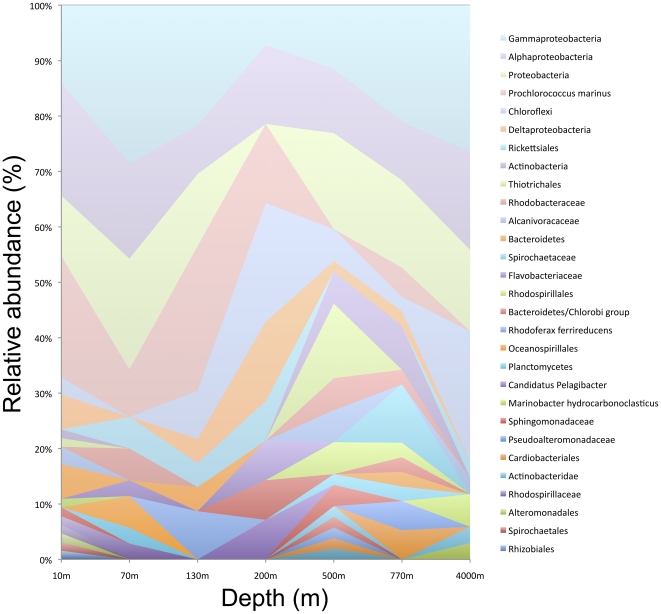
Relative abundance of metagenomic bacterial sequences from different taxonomic groups in samples along an oceanic depth gradient at the HOT ALOHA site, identified by phylotyping of sequences by AMPHORA[26]. Sequences that could not be placed reliably (phylotyping bootstrap <70%, only placed to ‘Bacteria’ level) were excluded.

Phylotyping of metagenomic sequences sheds further light on the patterns of taxonomic and phylogenetic diversity we observed along the depth gradient (Figure 4). Samples from the photic zone (10 m–150 m depth) were generally dominated by sequences assigned to a few clades of highly phylogenetically similar groups, in particular to *Prochlorococcus*. Samples from greater depths were dominated by a variety of groups including α- and β-proteobacteria and Chloroflexi. These differences in taxonomic composition explain the differences we observed between taxonomic and phylogenetic diversity versus depth. Taxonomic binning at a 95% or 99% sequence similarity cutoff is only able to detect overlap between samples when they share extremely phylogenetically similar sequences, such as the closely related *Prochlorococcus* sequences that occurred primarily in the shallowest samples. Thus, taxonomic diversity is driven primarily by the presence of these closely related sequences, whereas phylogenetic diversity detected the evolution of associations with photic and non-photic habitats at deeper phylogenetic levels. Taxonomic binning approaches to analyzing metagenomic data ignore ecological variation that occurs at a level deeper than the similarity cutoff being used for binning, and in this data set ecologically important differences among organisms occurred at different levels of sequence similarity than the commonly used 5% SSU-rRNA gene similarity cutoff.

Based on taxonomic binning of the SSU-rRNA gene, DeLong *et al.* [22] detected only a handful of sequences from the SAR11 or *Pelagibacter ubique* clades, and SSU-rRNA gene sequences from these clades occurred in samples from depths >500 m [22], which is surprising given the usual abundance of these organisms in shallow ocean habitats [37]. Conversely, based on phylotyping of the metagenomic data, we found that sequences assigned to the broader taxonomic groups containing SAR11, including the α-proteobacteria, Rickettsiales and *Pelagibacter*, were among the most abundant in samples at depths shallower than 200 m (Figure 4). In samples from 10 m–130 m depth, 2%–6% of the sequences detected had *Pelagibacter ubique* as their closest relative across 571 reference genomes (Table S1). Differences in community structure measured using the SSU-rRNA gene versus metagenomic gene families could be explained by amplification bias or copy number variation in the SSU-rRNA gene [15] or overall differences in phylogenetic signal among gene families [41]; future simulation and phylogenomic studies will be required to distinguish among these possibilities.

### Conclusions

In summary, our results highlight the utility of metagenomic data for studies of microbial phylogenetic diversity and community assembly along environmental gradients. By combining phylogenetic information across multiple phylogenetic marker gene families in a metagenomic data set, we demonstrate that microbial communities show strong and consistent patterns of diversity variation along environmental gradients, patterns that may not be captured by taxonomic measures of diversity or by diversity measures based on a single marker gene. Thus, our approach allows the study of microbial diversity and community structure based on metagenomic data without the need to make assumptions about how to bin sequences into taxonomic or functional groups. Given the increasing availability of fully sequenced genomes and massive metagenomic data sets from high-throughput sequencing projects, the ability to estimate phylogenetic diversity from these data sets offers the potential to greatly improve our understanding of patterns of microbial diversity. A phylogenetic approach to measuring microbial diversity will make it possible to move beyond traditional taxonomic classification, towards understanding where on the tree of life habitat differentiation and adaptation are taking place.

## Methods

### Sequence identification and alignment

We analyzed a publicly available data set of DNA sequences from microbial communities in seven oceanic water samples along a depth gradient from 10 m to 4000 m depth at the HOT ALOHA site in the tropical Pacific [22]. This data set is comprised of both ribosomal DNA sequences obtained by PCR amplification and sequencing of the 16S SSU-rRNA gene (419 sequences total) and metagenomic sequences obtained by environmental shotgun sequencing of a large-insert fosmid clone library (64 Mbp of sequences total from the same environmental samples as the SSU-rRNA sequences). Analysis of the metagenomic data with the STAP pipeline [42] identified 30 bacterial SSU-rRNA gene sequences, which was insufficient to permit diversity analyses. We obtained the data, including unaligned SSU-rRNA gene sequences and translated peptide sequences of ORFs predicted from metagenomic reads, from CAMERA (http://camera.calit2.net/; CAMERA dataset node ID 1055661998626308448). Our taxonomic diversity analyses were based on analysis of the SSU-rRNA gene sequences and our phylogenetic diversity analyses were based on analysis of both the SSU-rRNA gene sequences and metagenomic data.

The 419 SSU-rRNA gene sequences were aligned using the STAP rRNA gene alignment and taxonomy pipeline [42]. For the phylogenetic diversity analyses of metagenomic data, we used the AMPHORA pipeline [26] to identify and align sequences from 31 gene families in the metagenomic data set. AMPHORA uses a hidden Markov model trained on a reference database of 571 fully sequenced bacterial genomes to identify and align metagenomic reads belonging to 31 marker gene families chosen based on their universality, low copy number, phylogenetic signal, and low rates of horizontal gene transfer [26]. From the 449,086 ORFs identified from the 65,674 reads in the full metagenomic data set, 497 reads could be assigned to one of the 31 gene families in the AMPHORA reference database.

### Phylogenetic tree inference

Phylogenetic relationships among SSU-rRNA gene sequences were inferred using FastTree version 2.0.1 [43] with a GTR+G substitution model and pseudocount distance estimation. The resulting phylogenetic tree was used to estimate branch length distances separating sequences for OTU binning, as well as for analyses of SSU-rRNA gene phylogenetic diversity.

Phylogenetic tree inference for metagenomic sequences required a different approach due to the fact that metagenomic sequences were relatively fragmentary and non-overlapping compared to the full-length SSU-rRNA gene sequences. Using aligned reference and metagenomic sequences from the 31 AMPHORA gene families, we combined reference sequences with metagenomic reads into a single large alignment (Data Set S1). Reference sequences were concatenated across all gene families for the organisms included in the reference database, and metagenomic reads were tiled against this alignment. Phylogenetic relationships among metagenomic sequences were then inferred by placing metagenomic sequence on a well-supported reference phylogeny. First, we inferred the reference sequence genome phylogeny using RAxML version 7.2.2 [29] to carry out a maximum likelihood tree inference using a WAG+G model partitioned by gene family on the 571 reference sequences. We compared the likelihood of phylogenetic trees linking all reference sequences inferred with a partitioned model (separate substitution rate and G parameter estimation for each gene family) to a non-partitioned model. The reference phylogeny obtained with a partitioned model of evolution had a higher likelihood than the phylogeny obtained from a non-partitioned model, supporting the use of the partitioned model for all subsequent analyses (log-likelihood of partitioned model phylogeny = −1,968,521, log-likelihood of non-partitioned model phylogeny = −1,969,550, likelihood ratio test *P*<0.001).

We placed all metagenomic reads on the reference phylogeny using the single-sequence likelihood insertion heuristic implemented in RAxML version 7.2.2 [29], [44]. This algorithm places query sequences (metagenomic reads) onto the reference phylogeny by evaluating the likelihood of query sequence placement on each edge of the phylogeny with optimization of query sequence branch length under the partitioned evolutionary model used to generate the reference phylogeny. We created one phylogeny based on the maximum likelihood placement of metagenomic reads onto the reference phylogeny (Data Set S2). We also created a distribution of replicate phylogenies where sequence placement uncertainty on the reference phylogeny was evaluated using a phylogenetic bootstrap [45]. The bootstrap analysis was repeated 100 times, resulting in 100 likely trees generated by placing bootstrap-resampled sequences on the reference tree with probability weighted by bootstrap placement at each edge for each sequence. For all phylogenies, we then pruned reference sequences from the tree leaving only the metagenomic sequences. The resulting phylogeny containing only the metagenomic sequences was used for subsequent analyses. We repeated all analyses on the maximum likelihood metagenomic tree and across the 100 bootstrap trees.

There were some metagenomic sequences that were either highly divergent or poorly placed on the reference phylogeny, resulting in a relatively long branch length subtending the sequence or relatively high uncertainty in placement of the sequence on the reference phylogeny. To assess the effect of these sequences on our results, we repeated analyses with a more conservative set of sequences by dropping the sequences whose subtending branch length connecting them to the reference phylogeny was in the top 5th percentile of subtending branch lengths, as well as sequences with fewer than 50 unmasked amino acids. Using a more conservative set of sequences did not change the trends we observed.

### Diversity analyses

Taxonomic classification of the aligned SSU-rRNA gene sequences by the STAP rRNA alignment and taxonomy pipeline [42] indicated that 67 of 419 SSU-rRNA gene sequences were archaeal and 352 were bacterial. To allow direct comparison of taxonomic diversity with the metagenomic bacterial sequences identified by AMPHORA, we analyzed only the bacterial SSU-rRNA gene sequences. Including archaeal sequences in calculations of diversity did not have an effect on the trends we observed. Taxonomic diversity was estimated based on operational taxonomic unit (OTU) binning of bacterial SSU-rRNA gene sequences at 95% and 99% similarity cutoffs with a complete linkage algorithm using mothur version 1.6.0 [46] with distances among sequences based on the phylogeny linking all SSU-rRNA gene sequences. Based on the SSU-rRNA gene OTU data we calculated taxonomic richness (the number of OTUs) for each environmental sample. Phylotyping and taxonomic identification of metagenomic sequences were performed as part of the AMPHORA algorithm, which places each sequence onto the reference bacterial genome phylogeny and determines taxonomic affiliation based on the NCBI taxonomy with bootstrapping to confirm confidence in taxonomic placement [26]. For subsequent analyses of taxonomic composition of the metagenomic sequences, we excluded 21% of the metagenomic sequences identified by AMPHORA that could be not be phylotyped with bootstrap support >70% to a taxonomic rank more precise than Bacteria.

We used the Picante software package [33] to calculate phylogenetic diversity within communities as the standardized effect size of mean pairwise phylogenetic distance (*SES_MPD_*) separating all pairs of sequences in each sample [1], [30]. *SES_MPD_* was calculated by simulation, based on a comparison of observed phylogenetic diversity with the phylogenetic diversity in 999 random draws of the observed number of sequences in a sample from the phylogeny including all metagenomic sequences. Standardized phylogenetic diversity was calculated for each sample based on the SSU-rRNA gene phylogeny, the maximum likelihood metagenomic phylogeny, and across the 100 replicate metagenomic phylogenies inferred with a phylogenetic bootstrap.

Relationships between diversity and depth were calculated for all diversity measures. We compared linear and quadratic regressions of taxonomic and phylogenetic diversity versus log_10_-transformed depth to determine how diversity varied with depth, and whether the diversity-depth relationship was linear or quadratic (i.e., unimodal). While sample sizes were too small to allow formal model comparisons for taxonomic diversity and phylogenetic diversity based on the maximum likelihood tree, for the bootstrap replicate phylogenies the quadratic model of the phylogenetic diversity - depth relationship was a more parsimonious fit to the observed data than the linear model (*SES_MPD_* versus log_10_(depth): AIC of quadratic model: 2171.9, AIC of linear model: 2256.3).

## Supporting Information

Table S1 **Relative abundances of sequences assigned to differenet outgroups on a reference phylogenetic tree by AMPHORA** [**26**] **for metagenomic sequences collected along an oceanic depth gradient at the HOT ALOHA site** [**22**] **.** Outgroups represent the reference sequence most closely related to each metagenomic sequence based on a phylogenetic placement of each sequence on a phylogeny based on 31 gene families from 571 fully sequenced bacterial genomes.
(PDF)

Data Set S1 **FASTA alignment file containing aligned AMPHORA** [**26**] **reference sequences and metagenomic sequences from 31 gene families along an oceanic depth gradient at the HOT ALOHA site** [**22**] **.**
(FASTA)

Data Set S2 **Newick-format phylogenetic tree linking metagenomic sequences from 31 gene families along an oceanic depth gradient at the HOT ALOHA site** [**22**] **.** The tree is the one that was identified as having the maximum likelihood by placing metagenomic reads on a reference phylogeny inferred with a WAG + G model partitioned by gene family in RAxML [29].
(NEWICK)
