# Supplementary material for: The Phylogenetic Diversity of Metagenomes
Source: PLoS One. 2011 Aug 31;6(8):e23214. doi: 10.1371/journal.pone.0023214 (PMC3166145; doi:10.1371/journal.pone.0023214)
Supplement: Table S1 — Relative abundances of sequences assigned to differenet outgroups on a reference phylogenetic tree by AMPHORA [26] for metagenomic sequences collected along an oceanic depth gradient at the HOT ALOHA site [22] . Outgroups represent the reference sequence most closely related to each metagenomic sequence based on a phylogenetic placement of each sequence on a phylogeny based on 31 gene families from 571 fully sequenced bacterial genomes. (PDF) [file pone.0023214.s001.pdf]

Supplemental Table 1. Relative abundances of sequences assigned to differenet outgroups on a reference phylogenetic tree by AMPHORA for metagenomic sequences collected along an oceanic depth gradient at the HOT ALOHA site. Outgroups represent the reference sequence most closely related to each metagenomic sequence based on a phylogenetic placement of each sequence on a phylogeny based on 31 gene families from 571 fully sequenced bacterial genomes.

Outgroup	10m	70m	130m	200m	500m	770m	4000m
Acetobacteraceae	0.00	0.00	0.00	0.00	0.01	0.01	0.00
Acholeplasmataceae	0.00	0.00	0.00	0.00	0.01	0.00	0.00
Acidobacteria	0.00	0.04	0.00	0.00	0.01	0.03	0.01
Acidobacteria bacterium Ellin345	0.00	0.00	0.00	0.02	0.00	0.01	0.01
Actinobacteria	0.01	0.00	0.00	0.00	0.00	0.01	0.00
Actinobacteridae	0.02	0.00	0.05	0.02	0.05	0.07	0.00
Actinomycetales	0.00	0.00	0.00	0.00	0.01	0.01	0.01
Alcanivorax borkumensis SK2	0.03	0.02	0.00	0.00	0.03	0.00	0.01
Alkalilimnicola ehrlichei MLHE-1	0.00	0.00	0.05	0.00	0.00	0.00	0.00
Alphaproteobacteria	0.02	0.06	0.00	0.04	0.06	0.02	0.04
Aquifex aeolicus VF5	0.02	0.00	0.00	0.00	0.00	0.03	0.00
Bacteria	0.02	0.04	0.12	0.09	0.07	0.13	0.10
Bacteroidetes/Chlorobi group	0.00	0.00	0.05	0.09	0.01	0.04	0.00
Bdellovibrio bacteriovorus HD100	0.00	0.00	0.00	0.07	0.01	0.01	0.00
Betaproteobacteria	0.00	0.00	0.00	0.02	0.00	0.01	0.00
Borrelia burgdorferi group	0.00	0.00	0.00	0.00	0.02	0.03	0.00
Burkholderiales	0.02	0.00	0.00	0.00	0.00	0.00	0.00
Candidatus Pelagibacter ubique HTCC1062	0.02	0.06	0.02	0.04	0.00	0.00	0.01
Candidatus Phytoplasma asteris	0.00	0.00	0.00	0.00	0.01	0.00	0.00
Candidatus Sulcia muelleri GWSS	0.01	0.00	0.02	0.00	0.00	0.00	0.01
canis group	0.02	0.00	0.00	0.00	0.00	0.00	0.00
Caulobacteraceae	0.01	0.02	0.00	0.00	0.00	0.00	0.00
Chlamydiales	0.00	0.00	0.00	0.07	0.01	0.00	0.00
Chlorobiaceae	0.00	0.04	0.02	0.02	0.00	0.02	0.00
Chloroflexaceae	0.02	0.00	0.00	0.00	0.00	0.00	0.00
Chloroflexi	0.03	0.00	0.07	0.02	0.01	0.02	0.09
Chromohalobacter salexigens DSM 3043	0.01	0.06	0.00	0.00	0.00	0.00	0.00
Clostridiaceae	0.01	0.00	0.00	0.00	0.00	0.00	0.00
Clostridium phytofermentans ISDg	0.00	0.00	0.00	0.02	0.00	0.00	0.00
Clostridium thermocellum ATCC 27405	0.00	0.00	0.00	0.00	0.00	0.01	0.00
Coxiella burnetii	0.01	0.04	0.00	0.00	0.00	0.01	0.00
Cyanobacteria	0.00	0.02	0.00	0.00	0.01	0.00	0.03
Cytophaga hutchinsonii ATCC 33406	0.00	0.02	0.00	0.00	0.00	0.01	0.00
Dehalococcoides	0.00	0.00	0.00	0.07	0.05	0.01	0.18
Deltaproteobacteria	0.00	0.00	0.00	0.04	0.00	0.02	0.00
Desulfitobacterium hafniense Y51	0.00	0.00	0.00	0.00	0.00	0.00	0.01
Desulfococcus oleovorans Hxd3	0.00	0.02	0.00	0.00	0.00	0.01	0.00
Desulfotalea psychrophila LSv54	0.00	0.00	0.00	0.00	0.00	0.01	0.01
Desulfovibrionaceae	0.00	0.00	0.00	0.00	0.02	0.03	0.00
Dichelobacter nodosus VCS1703A	0.00	0.04	0.00	0.00	0.00	0.06	0.00
Epsilonproteobacteria	0.01	0.02	0.00	0.00	0.02	0.01	0.00
Flavobacteriaceae	0.00	0.02	0.00	0.02	0.00	0.00	0.00
Flavobacteriales	0.05	0.00	0.00	0.00	0.00	0.00	0.00
Francisella tularensis	0.03	0.00	0.00	0.00	0.01	0.00	0.00
Gammaproteobacteria	0.01	0.11	0.00	0.00	0.08	0.00	0.09
Geobacter	0.00	0.00	0.00	0.00	0.00	0.00	0.01
Hahella chejuensis KCTC 2396	0.01	0.00	0.00	0.00	0.00	0.00	0.00
Halorhodospira halophila SL1	0.00	0.00	0.00	0.00	0.02	0.00	0.00
Herpetosiphon aurantiacus ATCC 23779	0.00	0.00	0.00	0.00	0.00	0.00	0.01
Hyphomonas neptunium ATCC 15444	0.00	0.00	0.00	0.00	0.01	0.00	0.00
Lactobacillus reuteri F275	0.00	0.00	0.00	0.00	0.00	0.00	0.01
Leptospira	0.00	0.06	0.00	0.00	0.06	0.01	0.00
Magnetospirillum magneticum AMB-1	0.00	0.02	0.00	0.00	0.00	0.00	0.01
Marinobacter aquaeolei VT8	0.00	0.00	0.00	0.00	0.00	0.01	0.03
Marinomonas sp. MWYL1	0.01	0.04	0.00	0.00	0.00	0.01	0.01
Mesorhizobium loti MAFF303099	0.00	0.00	0.00	0.00	0.01	0.00	0.00
Microbacteriaceae	0.00	0.00	0.00	0.00	0.01	0.00	0.00
Micrococcaceae	0.02	0.00	0.00	0.00	0.00	0.00	0.00
Mollicutes	0.00	0.00	0.00	0.04	0.00	0.02	0.00
Mycoplasma	0.02	0.00	0.00	0.07	0.00	0.02	0.00
Mycoplasma gallisepticum R	0.01	0.00	0.00	0.00	0.00	0.00	0.00
Mycoplasma mobile 163K	0.00	0.00	0.00	0.02	0.00	0.00	0.00
Myxococcaceae	0.01	0.02	0.05	0.00	0.00	0.00	0.00
Myxococcales	0.01	0.00	0.05	0.00	0.01	0.00	0.00
Neorickettsia sennetsu str. Miyayama	0.01	0.02	0.07	0.00	0.00	0.00	0.00
Nitrosococcus oceani ATCC 19707	0.00	0.00	0.00	0.00	0.02	0.00	0.04
Oceanospirillales	0.00	0.02	0.00	0.00	0.00	0.00	0.01
Orientia tsutsugamushi Boryong	0.01	0.00	0.00	0.00	0.00	0.00	0.01
Parvibaculum lavamentivorans DS-1	0.02	0.00	0.00	0.00	0.00	0.00	0.00
Pasteurellaceae	0.00	0.00	0.02	0.00	0.00	0.00	0.00
Petrotoga mobilis SJ95	0.00	0.00	0.02	0.00	0.00	0.01	0.01
Prochlorococcus marinus	0.15	0.06	0.15	0.04	0.00	0.02	0.00
Prochlorococcus marinus str. MIT 9301	0.01	0.00	0.00	0.00	0.00	0.00	0.00
Propionibacterium acnes KPA171202	0.00	0.00	0.00	0.00	0.00	0.01	0.00
Proteobacteria	0.00	0.00	0.02	0.00	0.02	0.06	0.01
Pseudoalteromonas atlantica T6c	0.00	0.00	0.00	0.00	0.00	0.02	0.00
Psychrobacter	0.00	0.00	0.00	0.00	0.01	0.00	0.00
Rhizobiales	0.02	0.00	0.00	0.00	0.00	0.00	0.00
Rhodobacteraceae	0.02	0.00	0.00	0.00	0.06	0.02	0.01
Rhodoferax ferrireducens T118	0.00	0.00	0.05	0.00	0.00	0.00	0.00
Rhodopirellula baltica SH 1	0.00	0.02	0.02	0.02	0.03	0.01	0.01
Rhodospirillaceae	0.01	0.00	0.00	0.00	0.00	0.00	0.00

Rhodospirillales	0.00	0.00	0.00	0.00	0.02	0.01	0.00
Rhodospirillum rubrum ATCC 11170	0.03	0.04	0.00	0.00	0.00	0.00	0.01
Rickettsiales	0.01	0.00	0.07	0.00	0.01	0.02	0.00
Rickettsieae	0.00	0.00	0.00	0.00	0.00	0.01	0.00
Rubrobacter xylanophilus DSM 9941	0.01	0.02	0.00	0.00	0.03	0.00	0.00
Saccharophagus degradans 2-40	0.03	0.06	0.00	0.00	0.00	0.00	0.01
Salinibacter ruber DSM 13855	0.00	0.00	0.00	0.00	0.00	0.04	0.00
Silicibacter pomeroyi DSS-3	0.00	0.04	0.00	0.00	0.00	0.00	0.00
Solibacter usitatus Ellin6076	0.00	0.00	0.00	0.00	0.00	0.01	0.00
Sorangium cellulosum 'So ce 56'	0.02	0.00	0.00	0.00	0.00	0.03	0.00
Sphingomonadaceae	0.02	0.02	0.02	0.00	0.00	0.00	0.01
Sphingomonas wittichii RW1	0.00	0.00	0.00	0.00	0.02	0.00	0.00
Spirochaetaceae	0.00	0.00	0.02	0.02	0.00	0.00	0.00
Spirochaetales	0.00	0.00	0.00	0.02	0.00	0.01	0.01
Staphylococcus	0.00	0.00	0.00	0.02	0.00	0.00	0.00
sulfur-oxidizing symbionts	0.00	0.00	0.00	0.00	0.08	0.00	0.00
Syntrophomonas wolfei subsp. wolfei str. Goettingen	0.02	0.00	0.00	0.00	0.00	0.01	0.01
Syntrophus aciditrophicus SB	0.00	0.00	0.00	0.02	0.00	0.01	0.00
Thermotogaceae	0.02	0.00	0.00	0.02	0.00	0.01	0.01
Wigglesworthia glossinidia endosymbiont of Glossina brevipalpis	0.00	0.00	0.00	0.02	0.00	0.00	0.00
Xanthomonadaceae	0.01	0.00	0.00	0.00	0.01	0.02	0.03
Xanthomonas	0.01	0.00	0.00	0.00	0.00	0.00	0.00
